# Application of reverse vaccinology to design a multi-epitope subunit vaccine against a new strain of *Aeromonas veronii*

**DOI:** 10.1186/s43141-022-00391-8

**Published:** 2022-08-08

**Authors:** Sk Injamamul Islam, Moslema Jahan Mou, Saloa Sanjida

**Affiliations:** 1 Department of Fisheries and Marine Bioscience, Faculty of Biological Science, Jashore University of Science and Technology, Jashore, 7408 Bangladesh; 2 Center of Excellence in Fish Infectious Diseases (CE FID), Department of Veterinary Microbiology, Faculty of Veterinary Science, Chulalongkorn University, Bangkok, 10330 Thailand; 3 The International Graduate Program of Veterinary Science and Technology (VST), Department of Veterinary Microbiology, Faculty of Veterinary Science and Technology, Chulalongkorn University, Bangkok, 10330 Thailand; 4 Department of Genetic Engineering and Biotechnology, Faculty of Life and Earth Science, University of Rajshahi, Rajshahi, Bangladesh; 5 Department of Environmental Science and Technology, Faculty of Applied Science and Technology, Jashore University of Science and Technology, Jashore, 7408 Bangladesh

**Keywords:** *A. Veronii* TH0426, Vaccine, Epitopes, MD simulation, *E. coli* K12

## Abstract

**Background:**

*Aeromonas veronii* is one of the most common pathogens of freshwater fishes that cause sepsis and ulcers. There are increasing numbers of cases showing that it is a significant zoonotic and aquatic agent. Epidemiological studies have shown that *A. veronii* virulence and drug tolerance have both increased over the last few years as a result of epidemiological investigations. Cadaverine reverse transporter (CadB) and maltoporin (LamB protein) contribute to the virulence of *A. veronii* TH0426. TH0426 strain is currently showing severe cases on fish species, and its resistance against therapeutic has been increasing. Despite these devastating complications, there is still no effective cure or vaccine for this strain of *A.veronii*.

**Results:**

In this regard, an immunoinformatic method was used to generate an epitope-based vaccine against this pathogen. The immunodominant epitopes were identified using the CadB and LamB protein of *A. veronii*. The final constructed vaccine sequence was developed to be immunogenic, non-allergenic as well as have better solubility. Molecular dynamic simulation revealed significant binding stability and structural compactness. Finally, using *Escherichia coli* K12 as a model, codon optimization yielded ideal GC content and a higher CAI value, which was then included in the cloning vector pET2+ (a).

**Conclusion:**

Altogether, our outcomes imply that the proposed peptide vaccine might be a good option for *A. veronii* TH0426 prophylaxis.

## Background

*Aeromonas veronii*, a newly discovered aquatic pathogen, is also crucial for food safety and public health [1–3]. *A. veronii* can currently be isolated from almost any environment, and its prevalence in food and the environment may contribute to human illness [4–6]. Many infection pathways exist in *A. veronii*, posing unique challenges for the associated study [7]. Increasing numbers of patients have been infected with *A. veronii*, which has shown increasing virulence and drug resistance year after year. An increasingly large number of countries are now specifying *A. veronii* as a critical indicator of water quality because of its dangers. Additionally, as a result of mixed infections caused by *A. veronii* and other pathogens, septicemia has been a major problem in recent years in the aquaculture industry, posing a biosafety threat to humans [8]. The wide array of virulence factors present in *A. veronii*, as well as the complexity and diversity of its pathogenic pathways, create particular difficulties in research related to it [9]. However, there is no effective vaccine against *A. veronii* to combat its devastating effect on various fish species [10].

*Aeromonas veronii* TH0426, a new strain of *A. veronii*, exhibits enhanced virulence and adhesion due to cadaverine reverse transporter (CadB protein) and maltoporin (LamB protein) [9, 11, 12]. The LamB protein is a member of the gram-negative bacteria’s outer membrane porin family, which also includes the Maltoporin protein. The function of LamB protein has been studied in bacteria, the majority of which is *E. coli*. Previous research has shown that LamB protein is a λ phage receptor protein that also controls the content of maltose in fish body cells by transporting maltose and maltodextrin [13, 14]. At the same time, related studies suggested that as the LamB protein is a typical porins, the adhesion and internalization of it are important steps for pathogens to infect epithelial cells [15]. Moreover, wet lab analysis proved that the lamB gene of *A. veronii* plays a crucial role in the pathogenesis in various fish species [11]. Similarly, cadaverine metabolism is related to only one transport system, the lysine cadaverine reverse transport system. Cadaverine reverse transporter gene encodes a protein with 12 transmembrane helices, similar in structure to PotE protein from *E. coli*. The protein transfers lysine into the fish cell from the outside and excretes cadaverine [16]. Related research has found a link between the pathogenicity of pathogenic bacteria and the cadaverine reverse transport pathway, which is dominated by the Cad gene [17, 18]. The cadaverine reverse transport system increases pathogenicity of pathogenic bacteria directly or indirectly by boosting the transcriptional expression of virulence factors and influencing bacterial biofilm production, in addition to assisting pathogens in overcoming host pressure [19]. Moreover, experiment revealed that the cadB gene encoding the highly expressed protein CadB in *A. veronii* TH0426 strain and presence of cadB significantly enhanced the biofilm formation ability of *A. veronii* [9]. Ultimately, all these mechanisms cause serious infection and mortality in numerous fish species including Nile tilapia, rainbow trout, catfish, Japanese flounder, and sea bass.

The prompt discovery of safe, efficient, uncomplicated, economical, dependable, and fast development of immune induces against the guided antigen is made possible by the in silico design of multiepitope vaccines against microbial pathogens. Epitope-based vaccines have been successfully created in the postgenomic period to stimulate responsiveness against some of the worst human viruses, including chikungunya, ebola, influenza, Nipah, MARS-CoV, rota, and zika [20–25]. Previously, the in silico technique in fish had not been developed due to a lack of understanding of the differences between major histocompatibility complexes (MHC class I and II) and human leukocyte antigen (HLA) [26], but recent research on fish species has generated data to enable in silico techniques [26–29]. Already an in silico technique was effective in predicting epitopes and multi-epitopes with significant responsiveness against *Edwardsiella tarda*, *Flavobacterium columnarie*, *Vibrio harveyi*, marine birnavirus, and *Streptococcus agalactiae*, harmful pathogens in fish, separately [30–34].

MHC Class I, Class IIA, and Class IIB genes have been isolated and characterized in a wide range of fish species since Hashimoto et al. (1990) reported the first MHC genes in carp [35], including zebrafish [36], turbot [37], red sea bream [38], tongue sole [39], and Nile tilapia [40]. Both MHC class I and class II molecules were originated in the experimental data of the cord and tilapia for starting immune responses against infections. In this regard, the peptide with excellent binding capacities to HLA-A*0201, HLA-B*3501, and HLA-B*3508 might be employed as efficient vaccinations against certain fish diseases [26, 29]. In orange-spotted grouper and pompano, certain MHC Class IIB alleles have recently been found to be linked to viral and bacterial infections [41, 42]. The MHC IIB allele DBB*1001 was found to be strongly related to resistance to Singapore grouper iridovirus in orange-spotted grouper [43]. The DAB*01 allele was linked to immunity to *Photobacterium damselae* in pompano, while the DAB*04, DAB*05, and DAB*10 alleles were linked to *P. damselae* sensitivity [42]. In Nile tilapia *Oreochromis niloticus*, genetic variation in the major histocompatibility complex (MHC) Class IIB was investigated, as well as the relationship between MHC IIB alleles and disease resistance [44]. Resistance to *S. agalactiae* was found to be significantly connected to the alleles DAB*0107, DAB*0201, and DAB*0302, whereas susceptibility to *S. agalactiae* was found to be substantially linked to the allele DAB*0701 [44]. It was reported twenty-five MHC IIA alleles in Tilapia, among which DAA*1101 was significantly associated with Tilapia [44]. In addition, The *O. niloticus* genome was shown to contain at least 28 class I genes or gene fragments [45]. In Osteichthyes, a gene lineage of MHC class II molecules and three MHC class I molecules have previously been found [27]. Within the class Osteichthyes, which includes both marine and freshwater fish species, bony fishes are one of the most varied groups of vertebrates. Previous study reported that non-polymorphic MHC II sublineage E genes, which have a poor expression in immune system tissues, can be found in primitive fishes such as paddlefish, sturgeons, and spotted gar, as well as cyprinids, Atlantic salmon, European bass, channel catfish, turbot, and rainbow trout [46]. In fish, three MHC II molecule sublineages (MHC II-A, -B, and -E) have been found. The presence of MHC I-U and -Z lineage molecules has been demonstrated in zebrafish, Atlantic salmon, medaka, Nile tilapia, three-spined stickleback, spotted green pufferfish, and Mexican tetra, among other fish species [46]. In contrast, all of the MHC I and MHC II molecule lines, each with a distinct number of genes, were discovered only in Atlantic salmon. Furthermore, genotyping of MHC gene polymorphisms using targeted next-generation sequencing (NGS) technologies has recently been established for humans and certain nonhuman animals, and most species, including fish and crabs, have numerous highly similar MHC loci [27, 46, 47].

It is expected that in the coming days, computer-assisted techniques will be increasingly successful in controlling fish diseases [48, 49]. The final vaccine design implemented in this study also included in silico cloning, which could be used for future wet-lab synthesis and animal model testing. Engaged together based on computer-assisted techniques, the main objective of this research was to identify multiepitope from the best antigenic protein to fight motile aeromonads disease in fish species caused by *A. veronii*. Therefore, this study will further help the entire aquaculture sector to use vaccines against diseases and this novel approach will help the researcher to design a fast and effective vaccine for emerging diseases in fish.

## Methods

### Protein selection and allergenicity prediction

The NCBI database was used to retrieve *Aeromonas veronii* TH0426 cadaverine protein (CadB) (Accession No. UBR44598.1) and maltoporin (LamB) (Accession No. QHC09974.1) for antigen selection. The CadB and LamB protein sequences were obtained as FASTA files. VaxiJen v2.0 server was used to assess the protective antigens [50], and for each of them, a threshold value of 0.4 was chosen.

### Epitopes prediction

#### Epitope prediction and evaluation of cytotoxic T cell lymphocytes (CTLs)

The sequence of the chosen protein was entered into the CTLPred (http://crdd.osdd.net/raghava/ctlpred/) server to predict CTLs epitope [51]. The capacity of peptides to attach to MHC molecules was predicted using an artificial neural network (ANN) and a stabilized matrix method implementation (SMM). Epitopes were predicted based on previously reported alleles invertebrates. The predicted epitopes were further assessed through the VaxiJen v2.0 [50], MHC class I immunogenicity [52], ToxinPred [53], and AllerTop v2.0 [54] servers. All of the forecasts were made using the default parameters of each server.

#### Epitopes of linear B cell lymphocytes (LBL): prediction and evaluation

To promote humoral or antibody-mediated immunity, B cell epitopes are required. As a result, we used the iBCE-EL server with default parameters to predict the linear B cell lymphocyte (LBL) epitopes [55]. The anticipated epitopes were tested by using VaxiJen v2.0 and AllerTop v2.0 servers.

### Development of a multi-epitope vaccination

The vaccine was created by combining the chosen CTL and LBL epitopes with a suitable adjuvant and linking them with the proper linkers [56, 57]. Since glycoproteins recognize TLR5, and adjuvants are essential for overcoming the constraints of translation and synthesis, the adjuvant used here was TLR5 agonist [58, 59]. As a result, the adjuvant 50S ribosomal protein L7/L12 (NCBI ID: P9WHE3) was evaluated to boost the vaccine candidate’s immunogenicity. With the RS09 (APPHALS), the PADRE sequence (AKFVAAWTLKAAA) can break apart two b domains with weakly interacting interactions over a wide range of peptide lengths. The adjuvant RS09 is a synthetic agonist for the Toll-like receptor-5. It aids in the activation of both the innate and adaptive immune systems [60]. Innate immunity is activated by Toll-like receptors, as well as antigen presentation by antigen presenting cells (APCs). A PADRE sequence provides vaccine stability as well as adjuvant properties [61]. In contrast, the selected CTL was linked with the help of AAY linkers and the LBL was linked with the KK linker [56, 62]. The AAY linker is a proteasome cleavage site that has been exploited to modify protein stability, decrease immunogenicity, and improve epitope presentation [63]. The bi-lysine KK linker helps to maintain the separate immunogenic properties of the vaccine construct.

### Structural analysis of vaccine

The physiochemistry of a protein describes its fundamental characteristics. Physicochemical properties of the vaccine were predicted by the ProtParam server to gain a comprehensive understanding of the vaccine’s essential role [64]. We also evaluated the immunological properties through VaxiJen v2.0 [50], MHC-I immunogenicity [52], AllerTop [54], and SOLpro [65] servers. SOPMA (Self-Optimized Prediction Method with Alignment) server identifies the two-dimensional (2D) structural features of the construct, such as the α-helix, β-turn, and random coils [66] and PSIPRED v4.0 server [67] with default parameters. SOPMA has a prediction accuracy of above 80% [66]. To further understand the vaccine’s composition quality, 2D structural characteristics were retrieved and assessed.

### Prediction and confirmation of tertiary structure

The constructed vaccine was submitted to the RaptorX server (http://raptorx.uchicago.edu/) [68]. Using a cutting-edge algorithm and a 3D structure, the RaptorX server produces the most precise structure of the protein and its activities [68]. The C-score, TM-score value, RMSD, and top five models of a particular protein sequence may all be predicted and determined using this web service. The generated 3D structure was saved as a PDB file, which was chosen based on the C-score. The C-score on the server ranges from –5 to 2, with a higher number indicating a more confident protein model. For the refining of the vaccine structure, the discovered 3D structure was uploaded to the GalaxyRefine (http://galaxy.seoklab.org/refine) online web-based server. The CASP10 refining approach was used to operate this web server [69]. The RMSD, energy score, and overall quality score are all available on the GalaxyRefine website. The improved structure was downloaded, and the chosen structure was determined using the energy scores of the lowest and maximum RMSD values. PyMOL v2.3.4 was used to show the refined and discovered structure [70]. Analyzing the final 3D structure, we used the Ramachandran plot score (vaccine structure validity) and *Z*-score value, which indicates the standard deviation from the mean value. PROCHECK server was used to analyze Ramachandran plot, which runs most allowed and disallowed amino acid regions, and ProSA-web to analyze *Z*-score plot [71].

### Discontinuous B cell epitope prediction

B cell epitopes were found to be discontinuous in more than 90% of cases. A computer program ElliPro has been used to model the 3D structures of B cell epitopes that are discontinuous (conformational). Using the protrusion index (PI) values, ElliPro calculates three algorithms for determining the protein shape as an ellipsoid, the residue PI, and adjacent cluster residues. ElliPro calculates an average PI value for each output epitope, which is the sum of each epitope’s residues. The protein residues in the ellipsoid with a PI value of 0.9 are (90%) inside, while those outsides are (10%). The PI value for each epitope residue was determined based on its position outside of a maximum ellipsoid of residue mass. ElliPro is the best structure-based approach we have found for predicting epitopes with an AUC value of (0.837), the best of any protein prediction method we have tested.

### Disulfide engineering of the designed vaccine

To move forward and begin docking analysis, the designed model must be stable. Disulfide bonds provide a geometrically stable protein structure. Disulfide by Design 2.0 [72] was used to assign such bonds for the designed vaccine.

### Molecular docking

The binding interactions between modeled proteins and receptor molecules can be revealed through molecular docking experiments. For this, the ClusPro v2.0 server was used, which can be found at https://cluspro.bu.edu/, to submit the refined vaccine model as a ligand and the TLR5 protein as an immunological receptor for molecular docking [73]. The TLR5 receptor was chosen and downloaded from the PDB server (PDB ID: 3V44). Separating the associated ligand from the protein was the first step in preparing the receptor, which was followed by the removal of water and other chemicals. All of these procedures were carried out using the PyMOL v2.3.4 program [70]. Discovery Studio 2017 and PBDSum were used to investigate binding interactions and residues in the interacting surface. Online server ClusPro 2.0 was used for molecular docking and docking refinement, respectively. Again, the docking was performed for the third time using the HawkDock server and following that the Molecular Mechanics/Generalized Born Surface Area (MM-GBSA) score was calculated using the same server that forecasts the affinity score result, with the lowest prediction score deemed the better score.

### MD simulation

The binding stability of the selected candidate compounds towards the desired protein to the active site region of the protein was investigated using 50 ns molecular dynamic simulations (MDS) [74]. To analyze the thermodynamic stability of the receptor-ligand complex, the MDS of the receptor-ligand complex was performed using the “Desmond v6.3 Program” in Schrödinger 2020–2023 under the Linux framework [75]. A preset TIP3P water model was utilized to solve the system, with an orthorhombic periodic boundary box shape with a box distance of 10 Å allocated to both sides to maintain a certain volume. Following the construction of the solvated system with protein in complex with the ligand, the system has been minimized using OPLS_2005 force field parameters in addition to the standard protocol introduced in the Desmond module [75]. In protein preparation wizard, initially, protein preprocesses by adding hydrogens, creating disulfide bonds, filling in the missing side chains, and deleting waters using Epik (pH:7.0 ± 2.0) and optimizing by PROPKA pH: 7.0. In model system for simulation run, simulation time = 50 ns, trajectory intervals = 50 ps, total number of frames = 1000, ensemble class = NPT, temperature = 300 K, and one atmospheric (1.01325 bar) pressure. Finally, the simulation was carried out for 50 ns, and RMSF, RMSD, and protein secondary structure elements from the trajectories were analyzed to reveal the stability of the vaccine complex.

### Immune response simulation

Using the C-IMMSIM v10.1 server, the entire construct was uploaded for assessment of the vaccine’s potential immunological response [76]. As previously stated, we used a minimum gap of 30 days between two dosages in this situation [77]. One time step equals 8 h in real life, and three injections were administered in three separate simulations with 1-time step, 84 times, and 168 times, respectively. With the maximum simulation step value set to 100, all other stimulation parameters were left at their default settings.

### Codon adaptation and in silico cloning

To express a foreign gene in a host, codon optimization is necessary [78]. As a result, the construct was uploaded to the JCat service for codon adaptation (http://jcat.de/). We employed the commonly used *E. coli* K12 as the host in this study, and the entire procedure was carried out while avoiding the following three criteria: sites of restriction enzyme cleavage, binding sites of prokaryotic ribosomes, and rho-independent transcription termination. The codon adaptation index (CAI) value and guanine-cytosine (GC) concentration of the modified sequence were used to evaluate it [78]. Lastly, the in silico cloning of the adapted nucleotide sequence into the pET28a (+) expression vector was performed using the modified nucleotide sequence. SnapGene v4.2 software was used to carry out the entire in silico cloning procedure [79]. The RNAfold server was also used to assess the efficiency of translation and the thermodynamic stability of expressed mRNA sequences [80].

## Results

### Analysis of the proteins

We predict the subcellular location of these proteins and the transmembrane helices. Subcellular location is very important for developing drugs and vaccines as well as transmembrane helices. We predicted the subcellular location of CadB and LamB proteins in inner membrane and outer membrane, respectively. Furthermore, the transmembrane helices predicted to be 12 and 0 for CadB and LamB separately. Vaxijen server predicts the antigenicity of the proteins CadB (0.4532) and LamB (0.5721). In addition, the allergenicity and toxicity score of these proteins came out negative.

### Prediction of cytotoxic T cell (CTL) and linear B cell (LBL) epitopes

The selected target proteins were screened for CTL and LBL epitopes. In total, 127 distinct CTL epitopes for CadB and 197 for LamB with MHC-1 binding alleles were predicted. A list of the top three CTL epitopes for each protein which was non-toxic, non-allergenic, non-toxic, and immunogenic, was compiled (Table 1). Furthermore, 17 unique LBL epitopes were predicted for CadB and 19 epitopes for LamB epitopes based on their toxicity, immunogenicity, antigenicity, and non-allergenicity (Table 2). Among them, the top three epitopes with the best probability, antigenicity, allergenicity, and toxicity were chosen for final vaccine construction. The length of the epitope was selected at 12 (12-mer peptide).

**Table 1:** Final CTL epitopes used to construct MEBV

Protein name	Epitopes	C-score	Immunogenicity	Allergenicity	Antigenicity	Toxicity	Boman index kcal/mol
Cadaverine CadB	VSLIILMFY	1.5435	Positive	Negative	2.1554	Negative	2.83
	YSQNWNTTS	0.6854	Positive	Negative	1.0450	Negative	3.17
	GSDLHAVIS	0.5498	Positive	Negative	0.8453	Negative	2.89
Maltoporin LamB	NVNFLDLRY	1.3923	Positive	Negative	1.8491	Negative	2.54
	ESTTTVCNF	0.4172	Positive	Negative	0.6366	Negative	2.8
	GEDDNTWNF	0.3531	Positive	Negative	1.3355	Negative	3.76

**Table 2:** Final LBL epitopes used to construct MEBV

Protein name	Sequence	Probability	Antigenicity	Allergenicity	Toxicity
Cadaverine CadB	GLAFVFARLATK	0.5046	0.4945	Negative	Negative
	ATKNPQEGGPIA	0.7382	0.6242	Negative	Negative
	GEISPVFGFQTG	0.5653	0.8809	Negative	Negative
Maltoporin LamB	ALNANAVDFTGY	0.6653	0.6612	Negative	Negative
	NKDGKTFYVDSM	0.6734	0.9445	Negative	Negative
	DMQTGRQNFVGR	0.6609	0.9230	Negative	Negative

### The structure of a vaccine and its fundamental characteristics

**Fig. 1:**
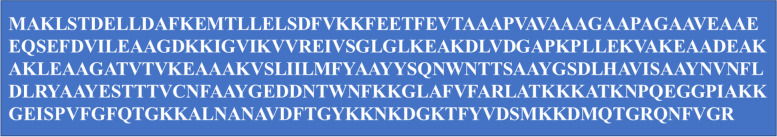
Constructed vaccine sequence

The vaccine was created utilizing epitopes from two distinct classes (CTL and LBL) that had previously been chosen. AAY and KK linkers were used to link epitopes together. To enhance immunogenicity, an adjuvant was applied before the construct. An adjuvant was attached to the CTL epitope with the RS09 (APPHALS) and PADRE sequence as a linker to activate TLR5 by ribosomal protein the 50S/L12 as an agonist. The final vaccination had a length of 288 amino acids (Fig. 1).

### Immunological assessment and physicochemical characteristics

**Fig. 2:**
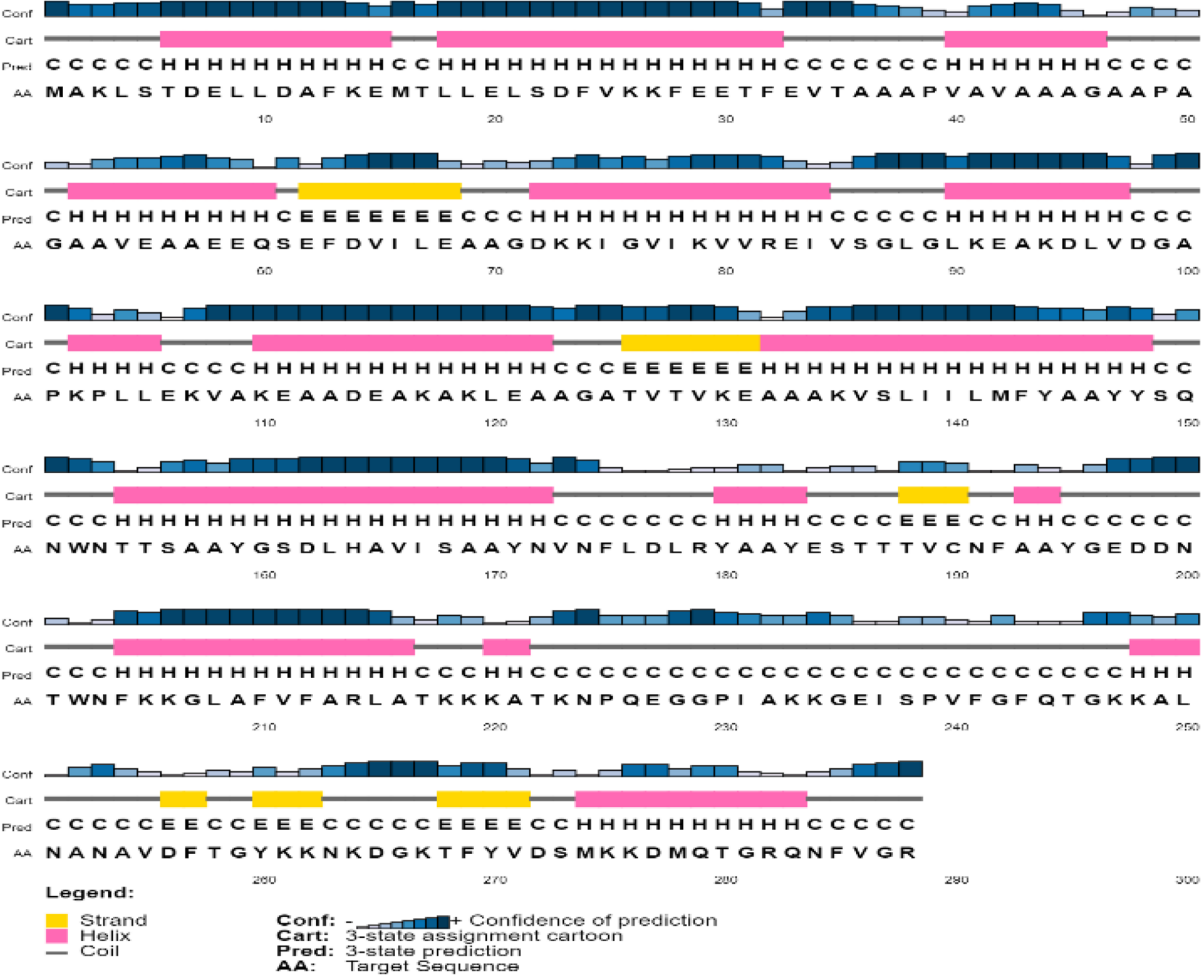
Prediction of the secondary structure of designed multi-epitope vaccines using PSI-PRED

Table 3 shows the physicochemical parameters of the vaccine construct. The construct was discovered to have a molecular weight of 30,890.22 Da. Other features such as the theoretical isoelectric point (pI) of 6.07, the chemical formula of C_1392_H_2193_N_357_O_423_S_6_, the instability index of 19.90, the aliphatic index of 81.15, and the grand average of hydropathicity of −0.135 were also present. The construct’s physicochemical properties and immunological efficacy were also assessed. For example, the construct’s antigenicity was 0.5828, whereas its immunogenicity was positive. Moreover, with a score of 0.87197 out of 1, the vaccine proved soluble (Table 3). α-helix, β-strand, and random coils were examined utilizing two distinct servers as secondary structural characteristics (Table 4). On the other hand, the PSIPRED server anticipated the features as 51.736% α-helix, 8.681% β-strand, and 39.583% random coils (Table 4) (Fig. 2).

**Table 3:** Characteristics of the construct in terms of antigenicity, allergy, and physicochemical properties

Characteristics	Finding	Remark
Number of amino acids	288	Suitable
Molecular weight	30890.22	Average
Theoretical pI	6.07	Acidic
Chemical formula	C_1392_H_2193_N_357_O_423_S_6_	-
Instability index of vaccine	19.90	Stable
Aliphatic index of vaccine	81.15	Thermostable
Grand average of hydropathicity (GRAVY)	−0.135	Hydrophilic
Antigenicity	0.5828	Antigenic
Immunogenicity	Positive	Immunogenic
Allergenicity	No	Non-allergen
Solubility	0.87197	Soluble

**Table 4:** Secondary structural properties of the vaccine

Characters	SOPMA		PSIPRED server	
	AA	%	AA	%
α-helix	151	52.43	149	51.736
β-strand	45	15.62	25	8.681
Random coil	70	24.31	114	39.583

### Refinement and confirmation of the tertiary structure

**Fig. 3:**
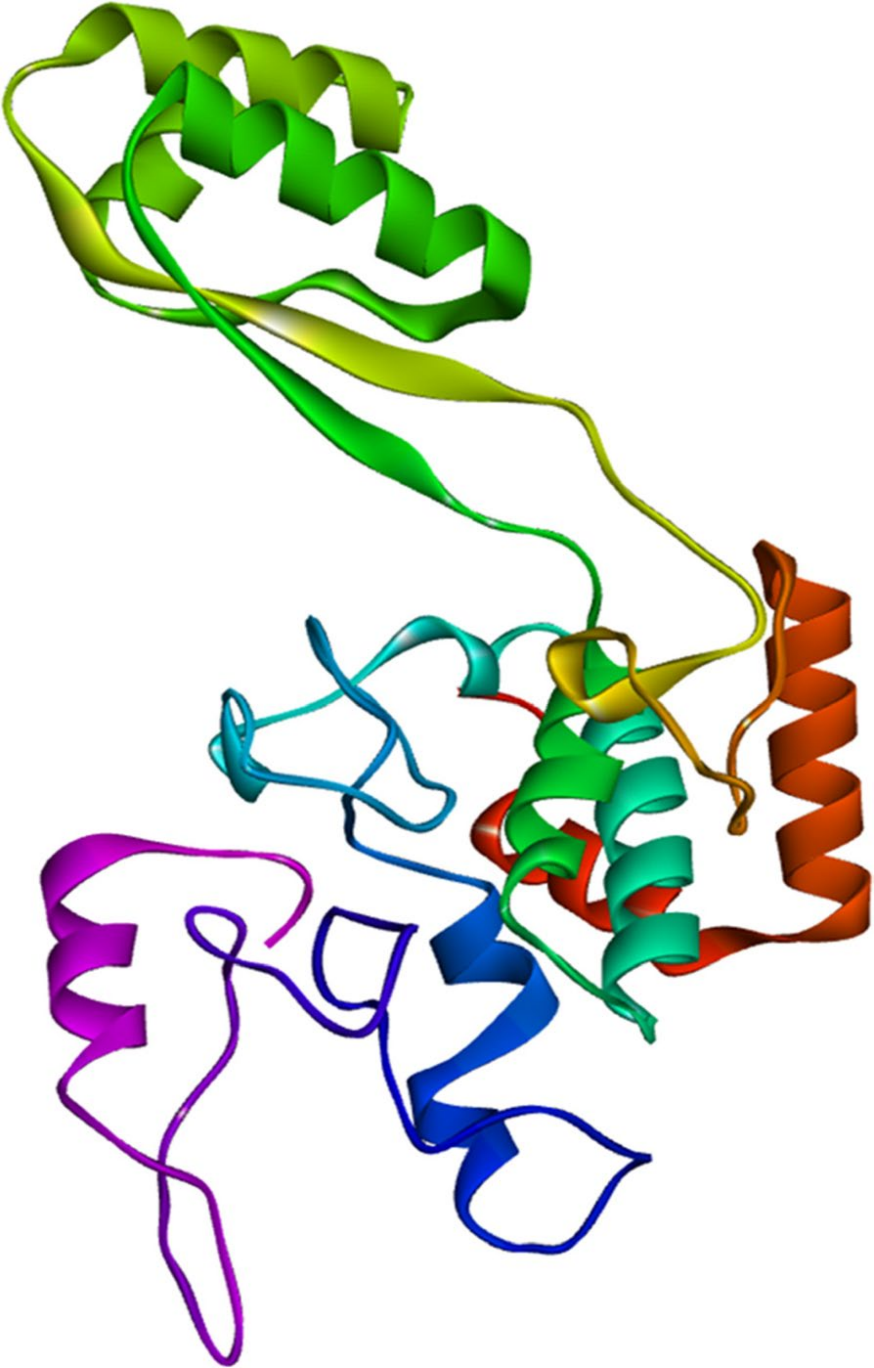
3D structure of the designed vaccine construct

**Fig. 4:**
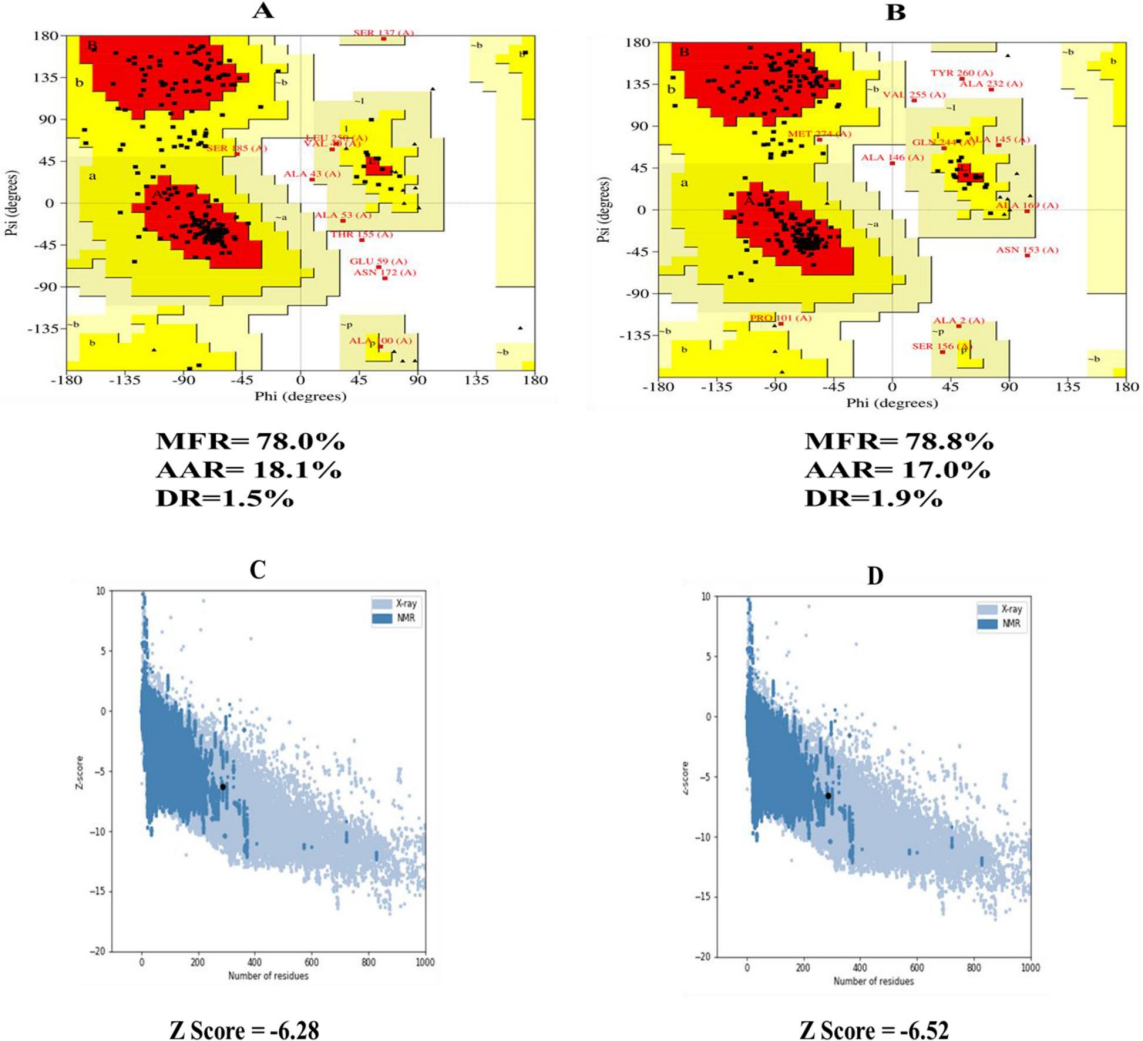
**A**, **B** Analysis of Ramachandran plot by PROCHECK server. **A** Ramachandran plot of the crude model of the vaccine and **B** Ramachandran plot of the refine model of the vaccine. The MFR, AAR, GAR, and DR have represented the most favored, additional allowed, generously allowed, and disallowed regions of the vaccine. **C** A 3D structure validation with a *Z*-score by Pro-SA server of the vaccine crude model. **D** A 3D structure validation with a *Z*-score by Pro-SA server of the vaccine refine model

The top five homology models were built using RaptorX as the best template. Out of the five models, we selected the one with the lowest C-score (–4.97). The 3D representation of the produced vaccine is shown in Fig. 3. Before refinement, the Ramachandran plot of the vaccine revealed that 78% of residues were in the most favorable zone, 18.1% in the additional allowed region, and 1.5% in the disallowed region. However, after refinement, 78.8% of the residues in the most favorable region were found in the Ramachandran plot, and 17% in additional allowed regions, while 1.9% in disallowed regions were seen Fig. 4B. The *Z*-score was −6.28 of the crude model whereas *Z*-score was –6.52 of the refine model (Fig. 4D).

### Conformational B cell epitope prediction

**Fig. 5:**
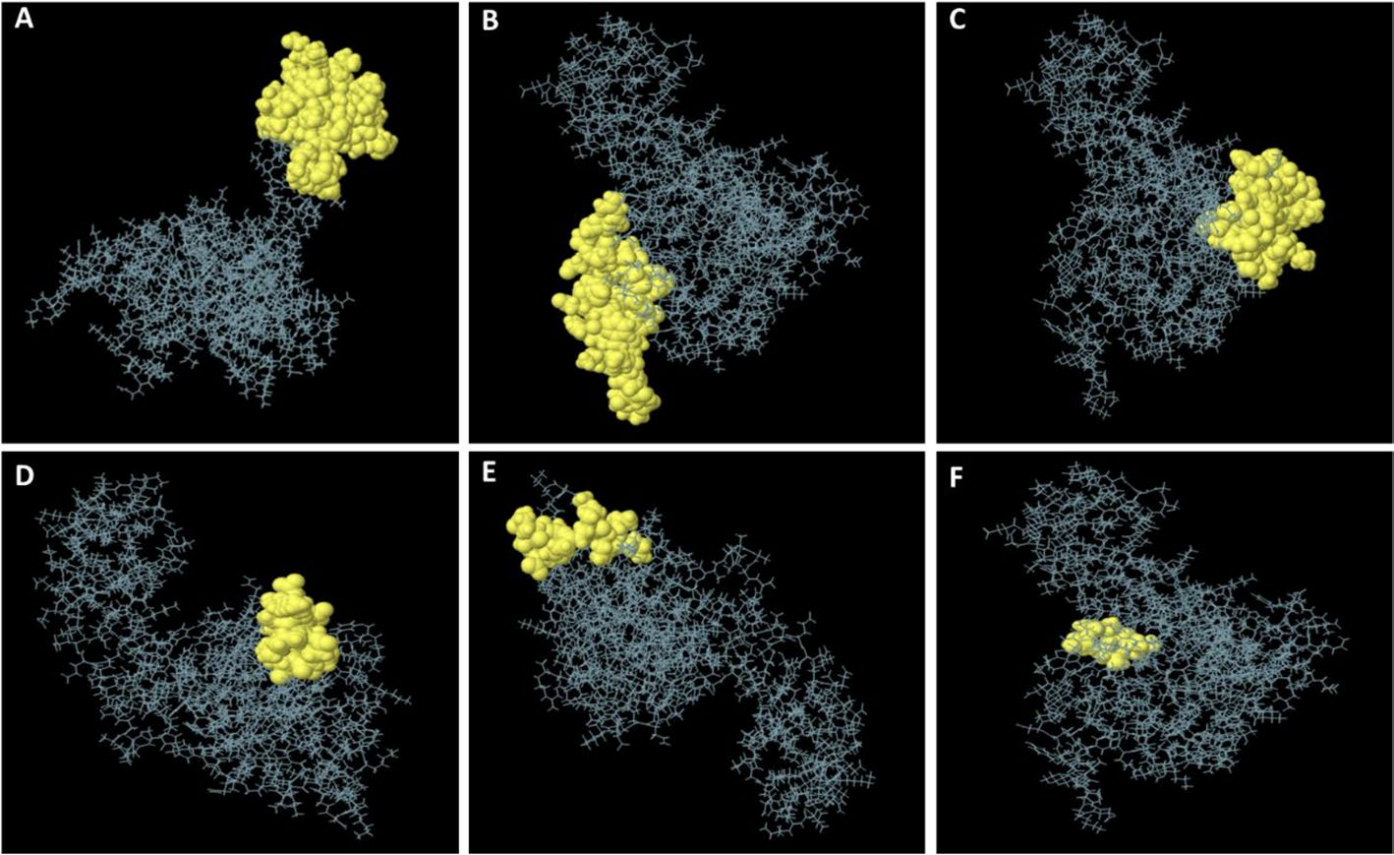
A three-dimensional representation of the developed epitope-based vaccine’s conformational or discontinuous B cell epitopes. **A**–**F** The conformational or discontinuous B cell epitopes are depicted by yellow surfaces, while the majority of the polyprotein is represented by grey sticks

**Fig. 6:**
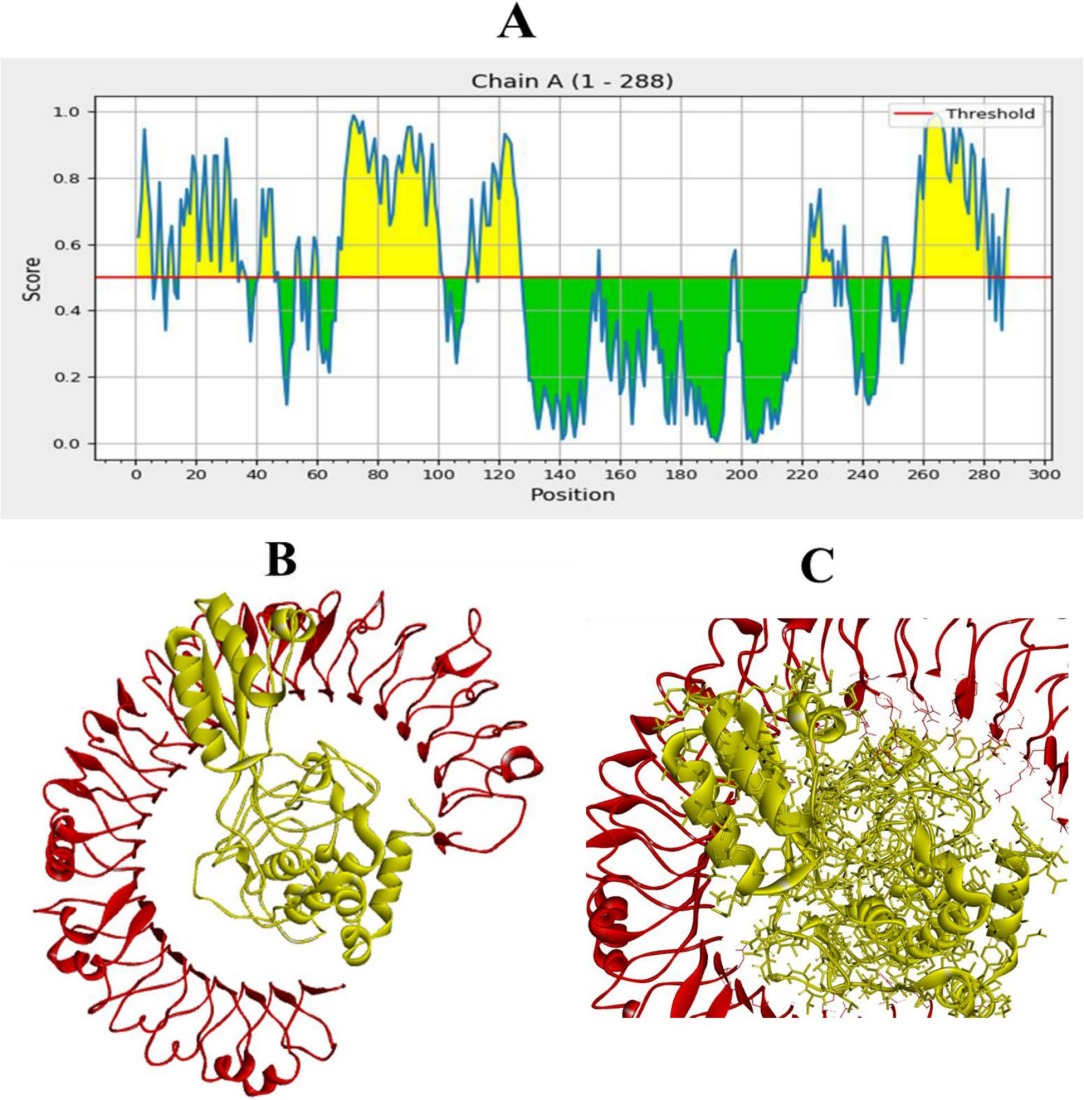
Discontinuous B cell epitopes and the ligand-protein (multi-epitope subunit vaccine)-receptor protein interaction (TLR-5). **A** Inside this epitope-based subunit vaccine, the specific value of discontinuous B cell epitopes was anticipated. **B**, **C** The ligand-protein is shown in yellow, whereas the receptor protein is highlighted in red

Four discontinuous B cell epitopes with values ranging from 0.568 to 0.779 were estimated to contain 143 residues. The size of the conformation epitopes varied from 6 to 55 residues. The score value of 0.568 or greater was chosen for discontinuous peptides that Ellipro predicted (Fig. 5A–F) and (Table 5). The individual score of each of the discontinuous epitopes from the vaccine sequence has been shown in Fig. 6A, and the ligand-protein interaction is shown in Fig. 6B–C.

**Table 5:** ElliPro predicted the conformational B cell epitopes residues of the designed epitope-based vaccine

No.	Residues	Number of residues	Score
1	A:L67, A:E68, A:A69, A:A70, A:G71, A:D72, A:K73, A:K74, A:I75, A:G76, A:V77, A:I78, A:K79, A:V80, A:V81, A:R82, A:E83, A:I84, A:V85, A:S86, A:G87, A:L88, A:G89, A:L90, A:K91, A:E92, A:A93, A:K94, A:D95, A:L96, A:V97, A:D98, A:G99, A:A100, A:P101, A:K102, A:V108, A:A109, A:K110, A:E111, A:A112, A:D114, A:E115, A:A116, A:K117, A:A118, A:K119, A:L120, A:E121, A:A122, A:A123, A:G124, A:A125, A:T126, A:V127	55	0.779
2	A:V240, A:A254, A:V255, A:D256, A:F257, A:T258, A:G259, A:Y260, A:K261, A:K262, A:N263, A:K264, A:D265, A:G266, A:K267, A:T268, A:F269, A:Y270, A:V271, A:D272, A:S273, A:M274, A:K275, A:K276, A:D277, A:M278, A:Q279, A:T280, A:G281, A:R282	30	0.763
3	A:D11, A:A12, A:E15, A:M16, A:T17, A:L18, A:L19, A:E20, A:L21, A:S22, A:D23, A:F24, A:V25, A:K26, A:K27, A:F28, A:E29, A:E30, A:T31, A:F32, A:V34, A:T35, A:A36, A:A37, A:P39, A:A52, A:A53, A:V54, A:E55	29	0.645
4	A:M1, A:A2, A:K3, A:L4, A:S5, A:D7, A:E8, A:L9, A:G196, A:E197, A:D198, A:D199	12	0.611
5	A:N224, A:P225, A:Q226, A:E227, A:G228, A:G229, A:P230, A:I231, A:A232, A:K233, A:K234	11	0.58
6	A:Q283, A:N284, A:F285, A:V286, A:G287, A:R288	6	0.568

### Vaccine disulfide engineering

To stabilize the vaccine design, disulfide engineering was employed. In the case of our vaccine, the DbD2 server found that there were 30 pairs of amino acids with the potential to make disulfide bonds. With other parameters such as energy and the chi^3^ value taken into account, two pairs of mutations with cysteine were recommended. Thus, SER37-CYS41 and ARG84-CYS105 were the residue pairs with the highest number of mutations. Energy and chi^3^ have approved values of less than 4.98 and 102.14: −113.64, correspondingly.

### Molecular docking research

**Fig. 7:**
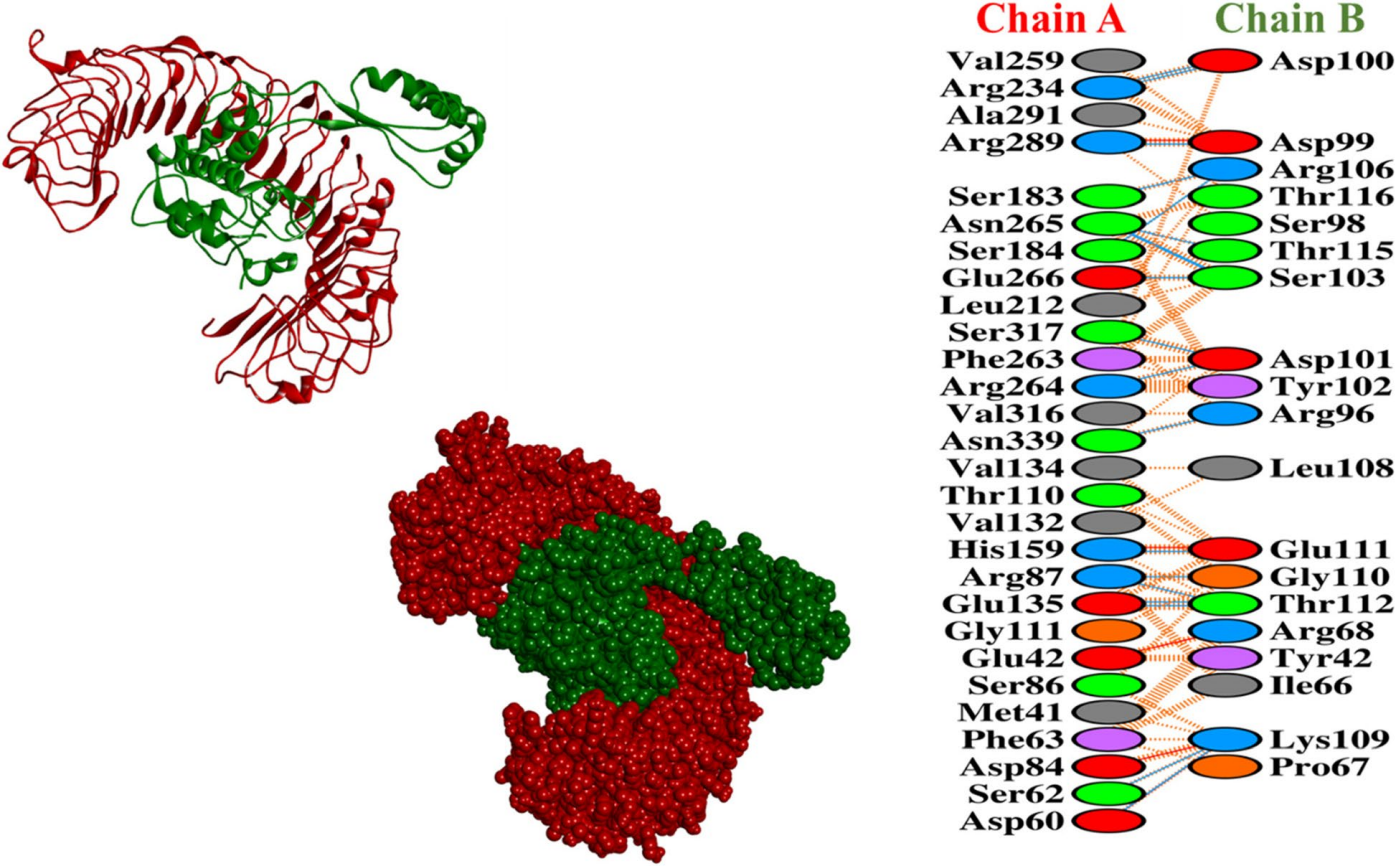
Analysis of EBV–immune receptor binding conformation and interaction. Intermolecular binding mode and residue-level chemical interactions of MEBV-TLR5

To predict their binding affinity and interactions, the vaccine (ligand) and TLR5 (receptor) were docked. As a result, the ClusPro v2.0 server produced ten docked complexes in various positions. We chose the complex with the lowest energy score and the binding posture with functional interactions from among them. As a result, model 1 met the inclination criterion. As a result, it was chosen as the best vaccine–TLR5 complex, with a –987.5 energy score. The PRODIGY function of the HADDOCK web-server was used to explore the docked complexes formed by ClusPro 2.0. The binding affinity score (kcal/mol) was calculated using the PRODIGY tool. HawkDock calculates the binding-free energy (kcal/mol) as well as ranking scores. After the MM-GBSA score on the HawkDock web server, the binding-free energy was calculated. When docked with TLR-5, the vaccine had the maximum binding affinity (37.72 kcal/mol) in the docking experiment conducted by the ClusPro 2.0 and PRODIGY servers. Furthermore, the vaccine showed the greatest results with the HawkDock server-nominated TLR-5, as well as in the MM-GBSA investigation, with a relative binding-free energy of 44.52 (kcal/mol). Binding interactions and residues implicated in active site residues were investigated in the chosen complex. A total of 16 hydrogen bonds were found on the interaction surface. The interacting residues in the CHB from the vaccine were Arg234-Asp100, Arg289-Asp99, Ser183-Arg106, Asn265-Arg106, Asn265-Thr115, Asn265-Ser103, Glu266-Ser103, Ser317-Asp101, Arg264-Asp101, Asn339-Arg96, His159-Glu111, Arg87-Gly110, Arg87-Thr112, Glu135-Thr112, Ser62-Lys109, and Asp60-Lys109. Moreover, associated TLR45 active site residues were shown in Fig. 7.

### Molecular dynamic (MD) simulation

**Fig. 8:**
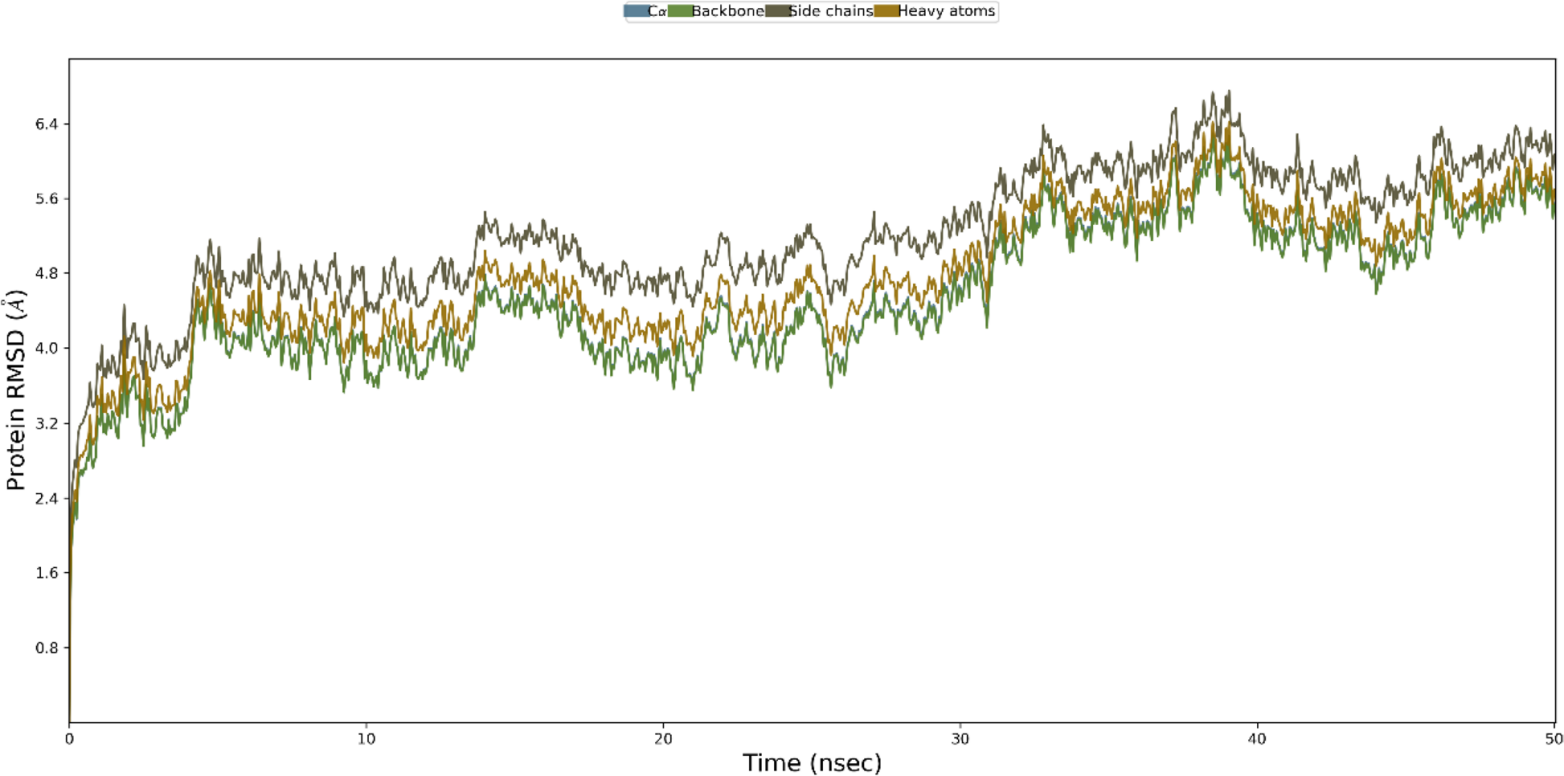
Simulation of the multi-epitope vaccine complex at the molecular level. The backbone atoms of the complexes were plotted using the RMSD method

**Fig. 9:**
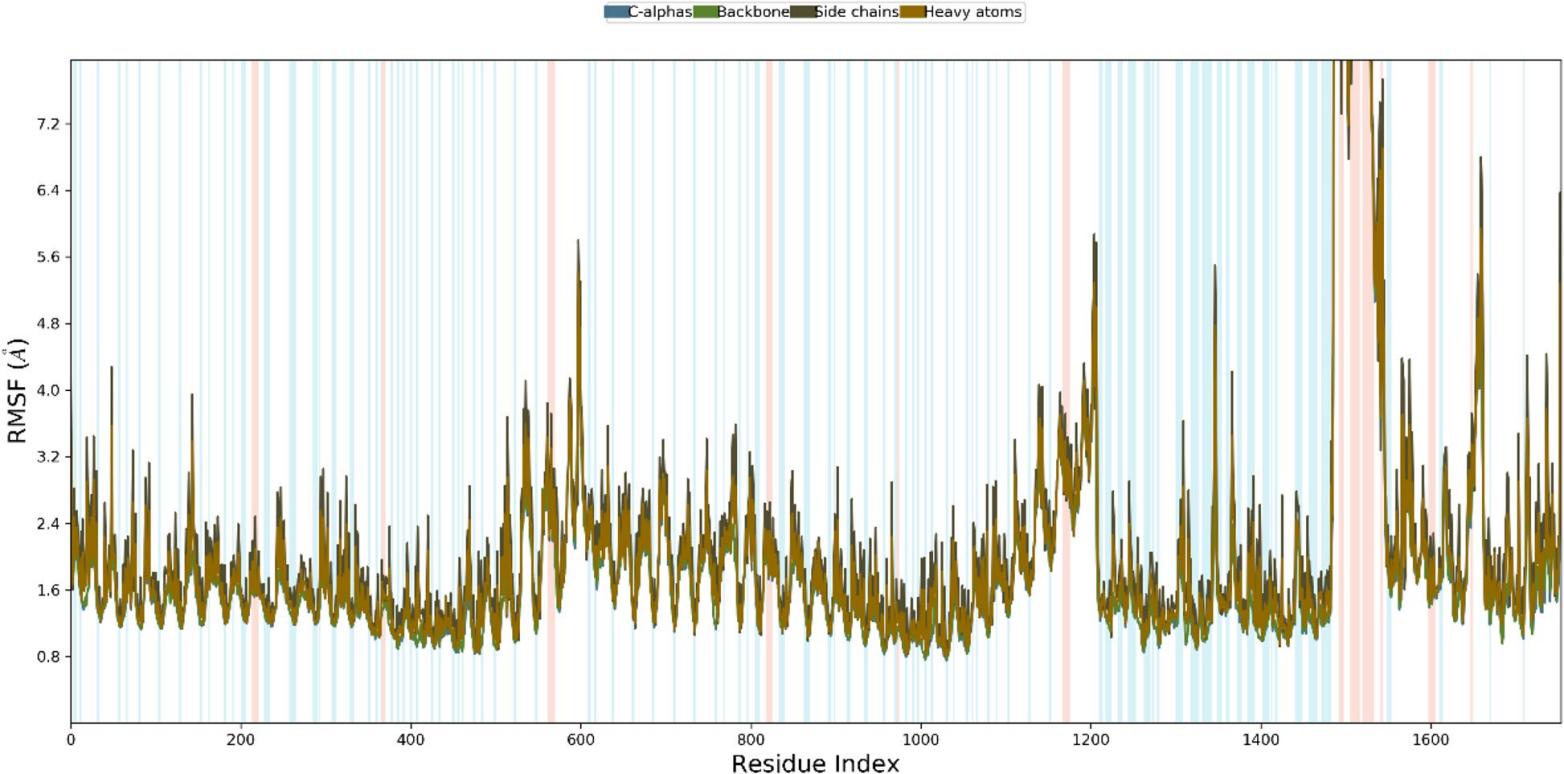
Simulation of the multi-epitope vaccine complex at the molecular level. The multi-epitope docked vaccine candidate RMSF plot. Red and blue backgrounds emphasize the alpha-helical and beta-strand sections, respectively. These areas are defined by helices or strands that last for 70% of the simulation time

The root mean square deviation (RMSD) of both the vaccine complex and vaccine was calculated. The average RMSD value for the vaccine complex was 4.88Å, which demonstrates the structural stability during the interaction. From Fig. 8, it can be observed that the vaccine complex has the initial increase of RMSD descriptors till 10 ns, and after that, it showed stability till 25 ns. A lower degree of fluctuation was observed from 7 to 8 ns, which may be responsible for structural integrity and/or to allow firm binding. Furthermore, the root mean square fluctuation (RMSF) score was used to assess protein flexibility across amino acid residues. The RMSF profile of the vaccine complex indicates maximum amino acid residues from complexes that an RMSF profile below 4.0 Å and greater change were observed for fewer residues. Figure 9 shows the stability and stiffness of the vaccine complex.

### Immune response simulation

**Fig. 10:**
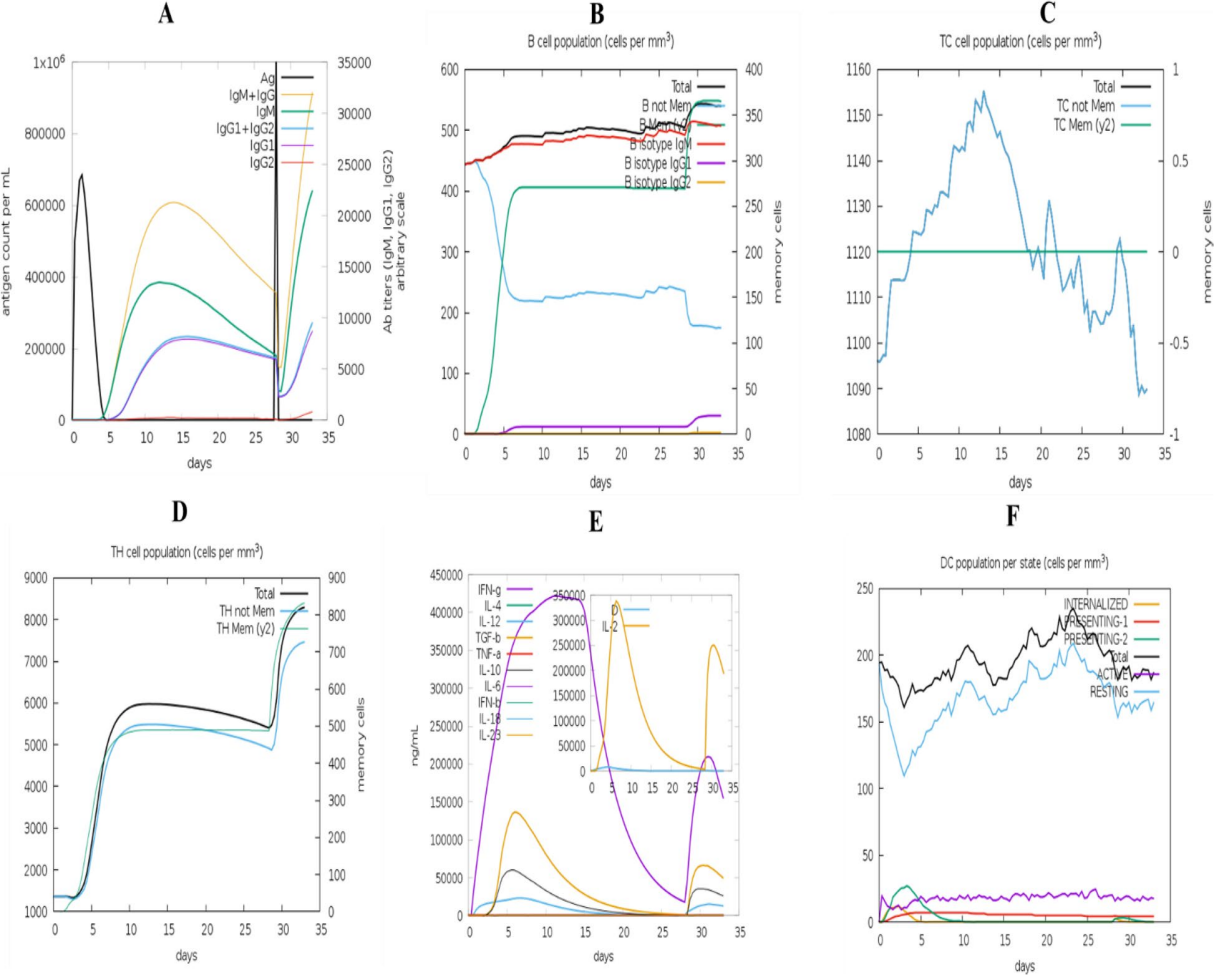
The vaccine has elicited an immune response. The graph shows **A** primary, secondary, and tertiary immune responses, **B** B cell population, **C** cytotoxic T cell population, **D** helper T cell population, **E** induction of cytokines and interleukins, and **F** dendritic cell population per state

As demonstrated in Fig. 10, the simulated immune response resembled genuine immunological events induced by certain infections. Secondary and tertiary immune responses, for instance, were greater than primary immunological responses (Fig. 10A). There were also secondary and tertiary responses, which were associated with higher antibody levels (IgG1+IgG2, IgM, and IgG+IgM), which led to significantly increased antigen clearance after subsequent exposures (Fig. 10A). In addition, B cells, cytotoxic T cells, and helper T cells had a longer survival time, indicating IgM memory development and class flipping between immune cells (Fig. 10B–D).

### Codon adaptation and in silico cloning

**Fig. 11:**
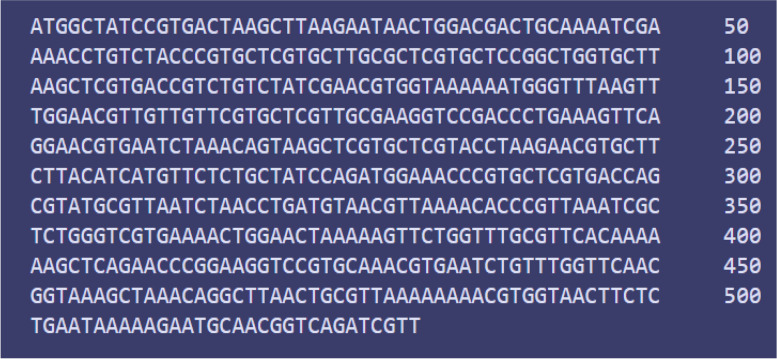
Codon adaptation of EBV to *E. coli* K12 strain

**Fig. 12:**
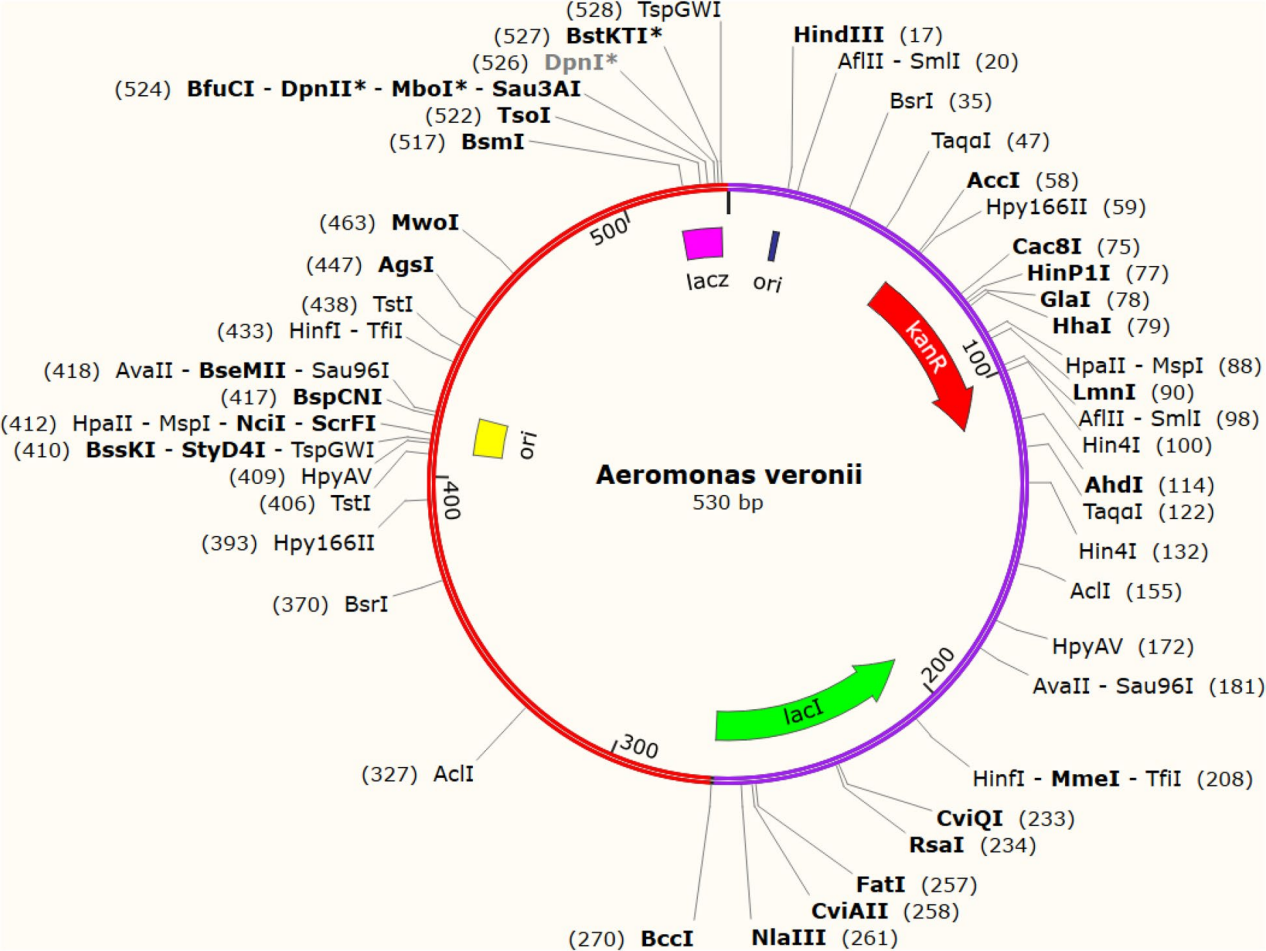
The proposed vaccine was cloned into the pET-28a (+) vector in silico

**Fig. 13:**
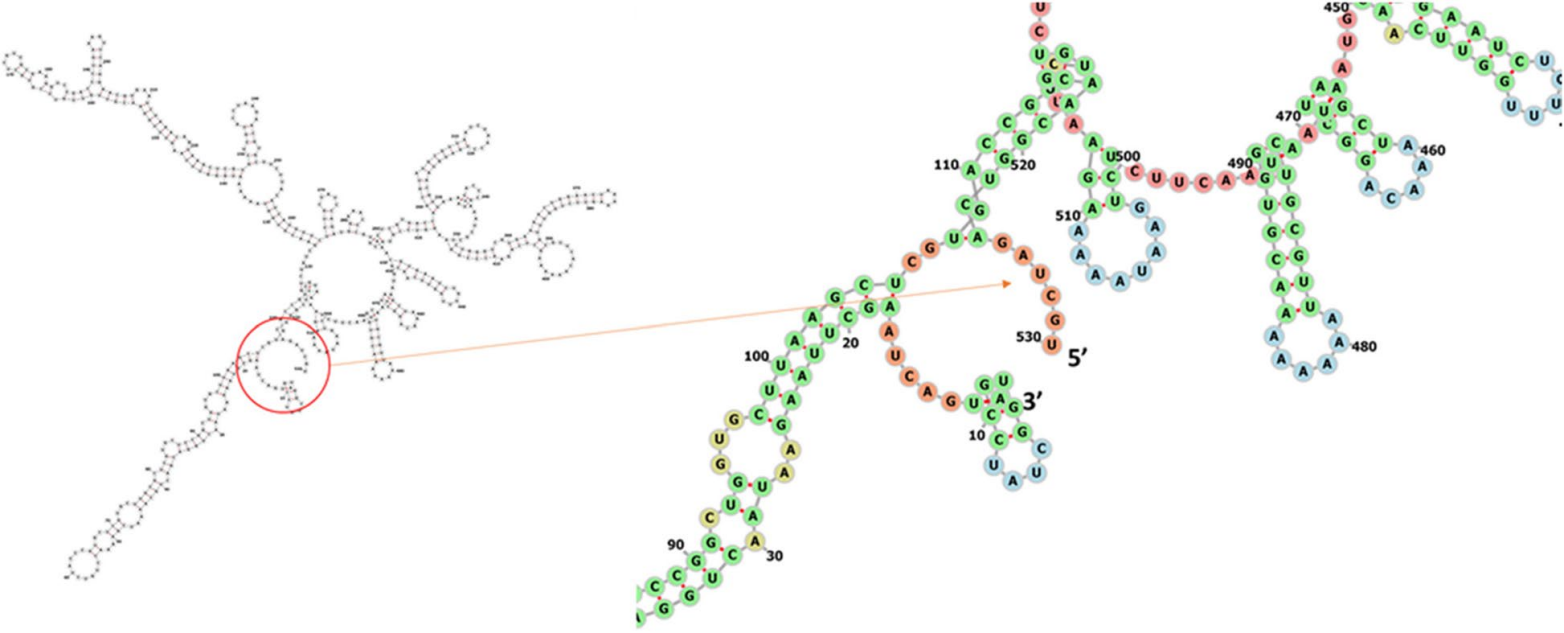
Predicted secondary structure of mRNA for the vaccine. The 5′ end of the predicted mRNA structure does not contain any pseudoknot or hairpin

To improve the translation efficiency of the vaccine design, we adjusted the codons according to the *E. coli* K12 on the JCat service. The nucleotide sequences created by the peptide vaccine construct (288 AA residues) totaled 530 lengths (Fig. 11). Furthermore, the modified nucleotide sequence has a GC content of 47.9647% and a CAI value of 1.0, respectively. We used the XhoI and BamHI restriction sites as the start and end cut points, respectively, to insert the altered sequence into the pET28a (+) vector. Using the SnapGene software, the modified vaccine construct was cloned into the pET28a (+) cloning vector (Fig. 12). The RNA fold server was used to predict the secondary structure of mRNA. The thermodynamic stability of the mRNA structure is indicated by the minimal free energy of −347.00 kcal/mol. Additionally, the initial 12 nucleotides of the mRNA secondary structure were free of any pseudoknots or long stable hairpins, enabling optimum translation initiation from the mRNA framework (Fig. 13).

## Discussion

Aquaculture throughout the world is currently vulnerable to infectious diseases caused by *Aeromonas veronii* [81, 82], which influences us to use an immunoinformatics method to build this epitope-based vaccine. The vaccination based on the virulent proteins displayed outstanding relevance as predicted by immunoinformatics in previous studies [33, 34, 49], proving our effort to be reliable. A vaccine protects against infectious illnesses safely and effectively [83]. Acquired immunity against contagious diseases should be possible with it [84]. *A. veronii* infection and transmission are difficult to control and prevent in the absence of an effective vaccine. Furthermore, to regulate the current situation, effective immunization has yet to be produced. As a result of this study, we designed a vaccine based on epitopes that would provide a strong immune response to *A. veronii* TH0426. There is a critical need for a novel vaccine development strategy to solve the economically threatening problem of aquaculture due to *A. veronii*.

Because the CadB and LamB of *A. veroni are* important for immunological invasion and transmission [9, 11], our goal was to develop an epitope vaccination that targeted the most virulent protein. enable cellular and humoral immune systems to recognize this protein, all the virulent protein selected through *in silico* screening was evaluated for its antigenic region. The first step was identifying all possible CTL and LBL epitopes [85]. Next, vaccines were designed with two antigenic epitopes—CTL and LBL, since the linkers below corresponded to the top epitopes. CTL and LBL epitopes were used in vaccine development as an important component that improves the stability, folding, and transcriptional regulation of our peptide vaccine [86]. Adjuvants are attached to CTL epitopes with RSO9 and PADREE sequences as linkers, which make the vaccine more stable and durable, as well as enhance cellular and humoral immunogenic responses [85, 87].

A total of 288 amino acid residues were found in the vaccine construction. An essential characteristic of a recombinant vaccine is its solubility, a type of physicochemical property [88]. A solubility assessing tool was used to determine whether the vaccine construct was solvable inside the host *E. coli*, and the results showed that it was solvable. The nature of the constructed vaccine, as indicated by the theoretical PI value, was basic. As recommended by server tools, the stability index of the vaccine sequence indicates that it will be stable following synthesis. The GRAVY value and aliphatic index, on the other hand, indicated that the vaccine was hydrophobic and thermostable, respectively. According to the prediction of physicochemical properties and scores on all parameters, there is a high probability for this vaccine to be a valid candidate against *A. veronii*. The detected models were revised and the best model (based on the lowest energy score) was chosen after the 3D structure prediction (based on *c*-score). We observed the *Z*-score (–6.52) and superior features of the most favored, acceptable, and prohibited areas for the Ramachandran plot in the validation test of the 3D structure. It was suggested by the lowest energy score of Vaccine-TLR5 complexes respectively, from a molecular docking suggested that the vaccine could have infection-inhibiting activity and might interact tightly with these receptors [34]. In Teleosts, TLR5 is sensitive to flagellin protein, so we employed flagellin peptides in epitope-based vaccine crafting, and it was found to function to induce inflammatory responses and develop innate immunity in fish [89, 90]. The molecular dynamics simulation is a potentially useful tool for understanding how proteins function and how their structure is derived [91]. Anatomical movement can be stimulated by protein dynamic simulations as a function of time. We have performed dynamic simulations of the vaccine candidate for 50 ns and analyzed the results using the RMSD and RMSF scores. When comparing distinct atomic conformations of a molecular system, the RMSD value is employed. Significant flexibility and departure of vaccine candidates from receptor structure were determined using the RMSD value, whereas the displacement of our particular vaccine candidate’s atoms from receptor structure was determined using RMSF of the complex structure. The calculated average RMSD and RMSF value was 4.88 Å and 4.0 Å, respectively. The fluctuation was not observed to be larger in the vaccine section, but it smoothed out after 5 ns, suggesting that the modeled vaccine and receptor are stable [33, 34].

Lastly, we examined the optimal target clearance and cell density parameters for the best immunologic response against the pathogen by constructing an immune simulation [92]. As a result of the upgraded vaccine doses, the immune system created memory B cells (with a half-life of several months) and T cells [34]. The vaccination efficiently imitated a humoral immune response to increased immunoglobulin production in this way. To optimize the multi-epitope vaccine production, the MD simulation was done to evaluate the stability of the vaccine candidate with the receptor, in which codon optimization was done for the stability of the construct vaccine within the host. Eventually, the codon was adjusted, and in silico cloning of the intended vaccine candidate into the *E. coli* K12 expression host *pET28a (+)* vector was successful.

## Conclusions

*Aeromonas veronii* TH0426 structures have been extensively studied, but control is still lacking due to its high mutation. A range of computational techniques was used in this work to find possible B and T cell epitopes in *A. veronii* virulent proteins, which were finally stitched into an epitope-based vaccine. Recently developed vaccine possesses the immuno-dominant qualities that are sought. Significantly, it was capable of binding to the immunological receptors and induces a substantial immune response in favor of bacterial infection. Based on our findings, we believe that preparing a vaccine against the etiological mediator of the *A. veronii* epidemic in fish should begin with the vaccine candidate. In addition, the possible epitopes discovered in this study can be employed in future research. Nevertheless, wet lab experiment is needed to show that our designed vaccine is effective against *A. veronii* TH0426.

## Data Availability

All data generated or analyzed during this study are included in this published article.

## Abbreviations

2D: Two dimensional
3D: Three dimensional
AAY: Ala-Ala-Tyr
Combined epitope: CTL and HTL epitopes
CTL: Cytotoxic T-lymphocyte
*E. coli*: *Escherichia coli*
GPGPG: Gly-Pro-Gly-Pro-Gly
JCAT: Java codon adaptation tool
KK: Lys-Lys
LBL: Linear B-lymphocyte
MD: Molecular dynamic
MHC-I: Major histocompatibility complex-I
MHC-II: Major histocompatibility complex-II
Rg: Radius of gyration
RMSD: Root-mean-square deviation
RMSF: Root mean square fluctuation
SOPMA: Self-optimized prediction method with alignment
TLR5: Toll-like receptor 5
